# COVID-19-associated invasive pulmonary aspergillosis

**DOI:** 10.1186/s13613-020-00686-4

**Published:** 2020-06-01

**Authors:** Lynn Rutsaert, Nicky Steinfort, Tine Van Hunsel, Peter Bomans, Reinout Naesens, Helena Mertes, Hilde Dits, Niels Van Regenmortel

**Affiliations:** 1 Dept. of Intensive Care Medicine, Ziekenhuisnetwerk Antwerpen, Campus Stuivenberg, Lange Beeldekensstraat 267, 2060 Antwerp, Belgium; 2 Dept. of Pneumology, Ziekenhuisnetwerk Antwerpen, Campus Stuivenberg, Lange Beeldekensstraat 267, 2060 Antwerp, Belgium; 3 Dept. of Medical Microbiology and Infection Prevention, Ziekenhuisnetwerk Antwerpen, Lindendreef 1, 2020 Antwerp, Belgium; 4 Dept. of Internal Medicine and Infectious Diseases, Ziekenhuisnetwerk Antwerpen, Campus Middelheim, Lindendreef 1, 2020 Antwerp, Belgium

To the Editor:

Since March 2020, following in the footsteps of China, Europe has been facing the COVID-19 pandemic, caused by the SARS-COV-2 virus [1]. Increasing numbers of patients are being admitted to intensive care units (ICU) throughout the world, imposing multiple diagnostic and therapeutic challenges on stressed healthcare systems. In our 24-bedded mixed ICU, we have encountered an unexpectedly high number of COVID-19 patients developing invasive pulmonary aspergillosis. Through our case series, we aim to raise awareness of this severe complication in the critical care community, point out different diagnostic obstacles and share our approach to the management of this complex problem.

Invasive pulmonary aspergillosis (IPA) is a well-known complication in immunocompromised patients and is encountered frequently in haematopoietic stem cell or solid organ transplant recipients [2]. Continued improvement in diagnostics has revealed that half of the cases of IPA occur in the ICU, in patients who are often non-neutropenic [3, 4]. Severe influenza infection is a well-known risk factor for developing IPA in non-neutropenic patients; a syndrome termed influenza-associated aspergillosis (IAA) [4–6]. A damaged respiratory epithelium, dysfunctional mucociliary clearance and a local immune paralysis were demonstrated to be key pathophysiological factors [4]. Supported by the hypothesis that alveolar damage facilitates fungal invasion, acute respiratory distress syndrome (ARDS) has frequently been associated with IPA in the ICU [6]. With this in mind, the existence of COVID-19-associated pulmonary aspergillosis is deemed likely.

Between March 12th and April 25th 2020, 34 COVID-19 patients were admitted to our ICU, of whom 20 (59%) required invasive mechanical ventilation. Seven of these ventilated patients (35%) were suspected of IPA (Table 1). Median age in our patient cohort was 66 (interquartile range 56–77) years. Underlying comorbidities were primarily cardiovascular. Only three patients were immunocompromised. One patient received chronic corticosteroid treatment for pemphigus foliaceous, one patient was HIV-positive (CD4 count > 250; viral load < 20 copies, treated with antiretrovirals [lamivudine/tenofovir/nevirapine]) and one patient had been treated for acute myeloid leukaemia 8 years ago and had developed IPA during chemotherapy. All patients were intubated and mechanically ventilated due to severe COVID-19 pneumonia.

**Table 1:** Patients with suspected IPA

Patient ID	Sex (M/F)	Age	Medical history	Time between COVID-19 symptoms and ICU admission (days)	Time between COVID-19 symptoms and intubation (days)	Time between intubation and first finding of IPA (days)	First BAL galactomannan index	Peak BAL galactomannan index	First serum galactomannan index	Peak serum galactomannan index
1	M	86	Hyperchol	7	7	9	N/A	N/A	0.10	0.10
2	M	38	Obesity, hyperchol	9	9	6	2.40	> 2.80	0.30	0.30
3	M	62	DM	7	7	16	0.72	2.00	0.20	0.20
4	M	73	DM, obesity, AHT, hyperchol	9	10	5	2.65	> 2.80	0.10	0.10
5	M	77	DM, CKD, AHT, pemphigus foliaceus	9	10	2	2.79	2.79	0.10	0.10
6	M	55	HIV, AHT, hyperchol	8	9	13	0.69	0.69	0.18	0.80
7	M	75	AML, IPA (2012)	3	3	8	2.63	2.63	N/A	N/A

Patient ID	Sex (M/F)	Age	Medical history	First positive culture	CT scan	Histological examination^a^	IPA diagnosis	AspICU algorithm	Antifungal therapy	Outcome
1	M	86	Hyperchol	*A. flavus* (ETA)	No scan available	N/A	–	–	None	Died, ICU day 10
2	M	38	Obesity, hyperchol	*A. fumigatus* (BAL)	+	Positive	Proven	Absolute	Voriconazole, isavuconazole	Alive on MV, ICU day 28^b^
3	M	62	DM	*A. fumigatus* (BAL)	No scan available	Positive	Proven	Absolute	Voriconazole	Died, ICU day 20
4	M	73	DM, obesity, AHT, hyperchol	*A. fumigatus* (BAL)	No scan available	Positive	Proven	Absolute	Voriconazole	Alive on MV, ICU day 24^b^
5	M	77	DM, CKD, AHT, pemphigus foliaceus	*A. fumigatus* (BAL)	No scan available	Positive	Proven	Absolute	Voriconazole	Alive on MV, ICU day 21^b^
6	M	55	HIV, AHT, hyperchol	*Negative*	No scan available	Negative		–	Voriconazole, isavuconazole	Died, ICU day 27
7	M	75	AML, IPA (2012)	*A. fumigatus* (BAL)	No scan available	N/A		–	Voriconazole	Died, ICU day 8

*CKD* chronic kidney disease, *AML* acute myeloid leukaemia, *IPA* invasive pulmonary aspergillosis. *N/A* no sample available, *Hyperchol* hypercholesterolaemia, *AHT* arterial hypertension, *DM* diabetes mellitus, *BAL* bronchoalveolar lavage, *ETA* endotracheal aspirate, *MV* mechanical ventilation arterial hypertension

^a^Anatomopathological examination of tissue samples obtained via bronchoscopy

^b^At the time of writing

Our suspicion was raised initially through an unusually rapid growth (< 48 h) of *Aspergillus* species in bronchial aspirates of three different patients. All samples were obtained during routine bronchoscopies, performed for atelectasis, respiratory deterioration or increasing inflammatory parameters. From that moment, routine galactomannan assays on serum and bronchoalveolar lavage (BAL) fluid were assessed regularly and bronchoscopy-guided biopsies of suspicious tracheobronchial lesions were obtained whenever present. Unfortunately, computed tomography (CT) scanning was deemed unfeasible in some patients due to extreme hypoxia or difficult mechanical ventilation and whenever performed, the distinction between COVID-19 and Aspergillus lesions proved complex.

Table 1 shows the timing and results of the microbiological testing in our case series. Differentiating between Aspergillus colonization and IPA is notoriously difficult, especially in the ICU. In the absence of host factors, as defined by the European Organisation for Research and Treatment of Cancer (EORTC) diagnostic criteria, invasive or high-risk diagnostics (biopsy, CT scan) are required to support the diagnosis of IPA [7]. The *AspICU* algorithm was designed to partially deal with the absence of host factors [6]. Based on this algorithm, four patients (No 2, 3, 4, 5) were diagnosed with proven IPA, based on histopathological evidence. All of these patients showed positive galactomannan indices on BAL fluid. In two patients, cultures and/or galactomannan BAL only became positive post mortem (No 1, 7), before CT scan or histopathological samples could be obtained. In one patient (No 6), histopathological sampling was negative and galactomannan BAL only mildly raised, but a raised serum galactomannan was later detected. In the remaining patients, the serum galactomannan index remained negative (< 0.5). The mean time between intubation date and the first microbiological signs of IPA was a striking 8 (SD 5) days. 

ICU physicians often have to weigh the risks of further diagnostic tests against a delayed initiation of antifungal treatment, which is associated with mortality rates over 65% [6]. Because all patients with clinical features of possible IPA were suffering from severe respiratory failure and hemodynamic instability, we initiated antifungal therapy as soon as cultures or galactomannan assays were positive. Five patients were started on voriconazole. In two of these patients, the treatment was escalated to isavuconazole due to pancytopenia or undetectable voriconazole levels under continuous renal replacement therapy. Two patients died on treatment.

To confirm and control this alarming incidence of COVID-19-associated IPA, a number of measures were taken. Firstly, we ruled out an environmental source, by sampling room air and the oxygen and pressurized air supplies (MAS 100, Merck). Prior to COVID-19, the incidence of IPA in our ICU was not elevated. Nonetheless, high-efficiency particulate air filters (HEPA) (Halton Vita, Helsinki, Finland) were installed in the ICU. Secondly, all mechanically ventilated COVID-19 patients were screened systematically by performing serum galactomannan assays twice weekly. Whenever a bronchoscopy was needed, BAL galactomannan indices and mould cultures were requested, regardless of the indication for bronchoscopy. Finally, we initiated prophylactic nebulization of 12.5 mg of liposomal amphotericin B (Ambisome^®^, Gilead, Foster City, USA) in every mechanically ventilated patient without an established diagnosis of IPA [8]. Since the implementation of these measures, we have not encountered any new cases of IPA at the time of writing.

## Conclusion

Using this case series, we would like to raise awareness about COVID-19-associated pulmonary aspergillosis, in view of its potential detrimental outcome. We believe that a low threshold for screening, prophylaxis and early antifungal treatment is of paramount importance, especially since different immunosuppressive therapies have been suggested to treat patients suffering from this alarming condition.

## Data Availability

The datasets used and/or analysed during the current study are available from the corresponding author on reasonable request.

## Abbreviations

COVID-19: Coronavirus disease 2019
SARS-COV-2 virus: Severe acute respiratory syndrome coronavirus 2
ICU: Intensive care unit
IPA: Invasive pulmonary aspergillosis
IAA: Influenza-associated aspergillosis
ARDS: Acute respiratory distress syndrome
h: Hour
BAL: Broncho-alveolar lavage
CT: Computed tomography
EORTC: European Organization for Research and Treatment of Cancer
AspICU: Aspergillosis intensive care unit
No: Number
SD: Standard deviation
HEPA: High-efficiency particulate air filters
